# Interactions between worms and malaria: Good worms or bad worms?

**DOI:** 10.1186/1475-2875-10-259

**Published:** 2011-09-12

**Authors:** Mathieu Nacher

**Affiliations:** 1 Centre d'investigation Clinique épidémiologie Clinique Antilles-Guyane, CIC EC Inserm CIE 802, Centre Hospitalier de Cayenne, French Guiana; 2 Equipe EA 35 93, Epidémiologie des parasitoses et mycoses tropicales, Université Antille-Guyane, Campus Saint Denis, Cayenne, French Guiana; 3 Department of Tropical Medicine, School of Public Health and Tropical Medicine, Tulane University, New Orleans, USA

## Abstract

In the past decade there have been an increasing number of studies on co-infections between worms and malaria. However, this increased interest has yielded results that have been at times conflicting and made it difficult to clearly grasp the outcome of this interaction. Despite the heterogeneity of study designs, reviewing the growing body of research may be synthesized into some broad trends: *Ascaris *emerges mostly as protective from malaria and its severe manifestations, whereas hookworm seems to increase malaria incidence. As efforts are made to de-worm populations in malaria endemic areas, there is still no clear picture of the impact these programmes have in terms of quantitative and qualitative changes in malaria.

## Background

In 1977, it was reported that malnourished patients heavily infected with *Ascaris lumbricoides *were free of malaria [1]. In 1978, the same team reported that piperazine treatment of patients with a heavy worm burden of *Ascaris *was followed by an increase in malaria attacks [2]. For the following 20 years, there were no other studies on the subject, perhaps for lack of connections between the fields of nutrition and malaria epidemiology or immunology. In the past decade, the topic was rediscovered, this time from an immunological angle. Depending on which publication one reads, it is not clear if the outcome of this interaction is beneficial [3], neutral [4] or harmful [5]. The studies on the interactions between worms and malaria in humans have been very different in design, in the age groups sampled, in the way the malaria outcome and the exposure to helminths were considered. It is, therefore, impossible to conduct a meta-analysis to settle the matter. Despite these difficulties, the present review aims to synthesize the growing number of studies that have explored the interactions between worms and malaria. Pubmed and google scholar searches were performed for malaria and any of the keywords worms, helminth, *Ascaris*, hookworm, *Trichuris*, or *Strongyloides*. This search identified published papers which were then studied for additional references.

### Studies in humans (Additional File 1, Table S1)

#### *Ascaris lumbricoides *and malaria

When it comes to coinfections between worms and malaria, *Ascaris lumbricoides *has been the most cited worm. Eight studies found that *Ascaris *was associated with a reduction of malaria (incidence, prevalence or reduction of parasitemia)[1,2,6-11], two studies found that *Ascaris *was associated with an increase in malaria prevalence [12,13], and two studies found no relation to malaria [4,14]. For cerebral malaria or renal failure, two studies identified *Ascaris lumbricoides *as the only individual worm associated with protection from severe malaria in adults [3,15]. One study observed an increase of severe malaria in *Ascaris*-infected children [16]. This study is often cited as an example that worms are bad for malaria, but the use of vomiting - which can be caused by *Ascaris*- as a definition criteria of severe malaria is problematic [17]. To summarize in even broader strokes, for *Ascaris*, 10 studies observed protection from malaria, three found an increase of malaria and two found no association at all with malaria. Another approach to the question compiled national ecologic data on worm prevalence, malaria incidence, climate, and GDP per capita, showed a ten-fold increase in malaria incidence in equatorial climates, low GDP, and *Ascaris lumbricoides *prevalence < 25% relative to prevalence ≥ 25% (submitted).

#### Hookworm and malaria

Hookworms seem to be associated with more malaria. There is a marked spatial overlap between hookworm infections and malaria in Africa [18]. Hookworm is the second most common GI nematode reported to have interactions with malaria. Although there was an apparent potentiation of the protection associated with *Ascaris*[3], results often differ from those of co-infections with *Ascaris lumbricoides*. Seven field studies, and one regression trees analysis of ecological data, finding increased malaria [9,11,12,15,19-21], whereas one study did not find any association between hookworms and malaria [4].

#### Pooled GI nematodes and malaria

Because of the assumption that the immune response to different helminths has a "stereotypical" profile, some studies pooled GI nematodes together. An increase in incidence was observed in three studies [22-24], a decrease in prevalence was observed in 1 study [25], and no association was observed with malaria in three studies [4,14,26]. Regarding malaria severity, three studies observed that GI nematodes were associated with protection from severe malaria [9,27,28]. The association between GI nematodes and protection from severe malaria remained after controlling for socioeconomic and environmental factors [29], and nutritional indicators. The quantification worm burden was proportionally associated with the level of protection from severe malaria, and the number of different GI nematodes present was both proportionally associated with protection from severe malaria [27] and increased incidence [22].

#### Schistosomiasis and malaria

There again four studies found different results. One study on *Schistosoma mansoni *found increased *falciparum *malaria incidence [30], whereas two studies on *Schistosoma haematobium *found protection from malaria: significantly lower parasitaemia, and lower incidence, although not significantly for the first [26], and decreased incidence in the second study [31], and one found no significant difference [32].

Prevalence is determined by the combination of incidence and infection duration. In clinical cases of malaria, treatment usually interrupts the natural course of the infection. The influence of helminths on the duration of a malaria episode in humans is not known. However, they seem to reduce some symptoms of malaria that can lead patients to seek treatment [33]. This may thus increase the overall duration of the presence of detectable malaria parasites in the bloodstream and potentially increase prevalence. But an increase in prevalence can also be due to increased incidence. Although it is not always clear in the publications whether incidence or prevalence is reported, overall five studies observed increased incidence in those with helminths, three observed decreased incidence and two found no difference. For prevalence, six studies found an increase in patients with intestinal worms, three found a decrease, and one found no difference. When looking at which worms are predominantly involved in these observations hookworm was associated with increased malaria incidence in two studies, and increased prevalence in five studies; in contrast *Ascaris *was reported to be associated with decreased incidence in two studies, and with decreased prevalence in three studies; one study found increased malaria incidence and another finds increased malaria prevalence in *Ascaris*-infected persons. Overall, there are thus some striking differences between *Ascaris lumbricoides *and hookworm. Whether these differences are only due to immunological factors, or if, as hypothesized elsewhere, hookworm-related anaemia leads to increased attractiveness for the vector [15] remains to be demonstrated. The findings in adults cannot easily be extrapolated to children < 5 years of age. Some studies suggest important age-related differences [6,32], whereas others show results that seem to mirror the results from adult studies [9].

Apart from variations of incidence or severity, gastrointestinal nematodes have also been related to malaria in intriguing ways that raise important evolutionary questions, however, the increase of gametocyte carriage in nematode-infected patients [34], the increased multiplicity of infection [35,36], and the relation between *Ascaris *fecundity and fever [37] first need confirmation [15].

Most studies involved *Plasmodium falciparum*, but a protective effect from *Plasmodium vivax *malaria was also observed in three studies [2,10,11].

### Hypothetical immunological mechanisms

Worms have different life histories but they bias the immune responses towards a stereotypical TH2/Treg response [38]. Malaria immunity has been studied extensively but it is still incompletely understood [39]. The balance between the pro and anti-inflammatory cytokines seems central in the pathogenesis of severe malaria, but helminth infections, a major reality in the tropics, are rarely considered when immunity to malaria is discussed. Chronic helminth immune modulation towards TH2/Treg lymphocytes would therefore be presumed to modify a number of aspects of malaria immunity. But knowledge on this is still very sketchy, to say the least. One of the specific hypotheses for the association of worms with protection from severe malaria was that worms led to an increase in IgE complexes that activated the FCεRII (CD23) and thereby releases the anti-inflammatory IL10 and activated the inducible Nitric oxide synthase, which led to the release of nitric oxide, and reduced sequestration of parasitized red blood cells [40]. Another potential explanation for the observed interactions is that T cells with a regulatory function may be preferably induced in helminth-infected patients thereby leading to a suppression of TH1 cells and proinflammatory activity [41,42].

Another hypothesis is that worms decrease cytophilic IgG1 and IgG3 antibodies whereas it increased the non-cytophilic IgG2, IgG4, IgM antibodies, and that this altered antibody-dependent cellular inhibition (ADCI). This hypothesis leads to different predictions with both an increase in incidence and severity [5,43]. Although so far the findings seem to fit with the initial hypothesis, it does not prove that the reason why malaria incidence increased was the shift towards non-cytophilic immunoglobulins. The above hypotheses explore different aspects of the immune response and do not exclude one another.

### Same, but different

All studies on worms and their effect on the immune response against malaria parasites have pooled different worm species into one single variable. This was based on the premise that worms induce similar immune responses and the fact that sample sizes in these studies were too small for a detailed analysis of each worm species' individual effect. The epidemiological studies, however, seem to suggest that there are differences between the different GI nematodes. *Ascaris lumbricoides *seems to be singled out when the outcome is "less malaria", whereas hookworm seemed more frequently involved when increased incidence is studied. *Ascaris lumbricoides *lays more eggs and it is arguable that this may make it easier to detect. However, if immunity alone is considered, *A. lumbricoides *is the largest nematode and the weight of foreign biological material involved in a host is arguably larger than for any other parasite. Moreover, in laboratories around the world *Ascaris *antigens are notoriously potent allergens. A large field study comparing IgE concentrations between different GI nematodes showed that total IgE levels were highest in *Ascaris-*infected children [44]. The reason why this parasite would evolve to stimulate such intense reactions may be related to the potential acuteness of the surgical complications of *A. lumbricoides *and the adaptiveness of generating immune responses limiting superinfection. This would fit with the IgE CD23/NO hypothesis discussed above, and possibly with the Treg hypothesis. Although some GI nematodes lead to a skew towards non cytophilic antibodies, a study seemed to show that *Ascaris-*infected persons had higher total IgG1 and IgG3 concentrations [45].

The hypothetical mechanisms for the apparent singular increase of malaria in hookworm infections could be linked to the combination of immune modulation and hookworm-related blood loss, which could, depending on the level of anemia, increase cues that are attractive for the vector (lactates, increased respiratory frequency and CO2 exhalation, increased cardiac output) thus leading to a greater probability of infective bites [15]. In this perspective, the hookworm vaccine initiative could have a interesting double impact: protection from hookworm's direct morbidity and maybe it's impact on malaria incidence.

### Animal models

Animal models have been use to explore co-infections between different helminths and malaria parasites before the first studies in humans [46]. Additional File 2, Table S2 summarizes the various experiments that have been conducted since then. The malaria outcomes of the interaction range from protection (13 studies) to increased severity (eight studies), while other models found no effect (5 studies). It is difficult to get a simple general idea of the outcome of the different coinfections. It should be emphasized that these models are not natural infections and therefore natural selection has not operated on outcome of repeated interactions. In addition, the extrapolation of findings on murine cerebral malaria to human cerebral malaria is questionable [47]. The insights gained from the models thus seem difficult to apply to humans.

## Conclusions

The default condition of the mammalian ancestral immune system was to be parasitized by gastrointestinal nematodes and other helminths, and acute responses to microparasites were tuned to this TH2/Treg skew. Researchers have long been unaware of the fact that worms significantly impact malaria, the strongest known selective force on the human genome. Despite a growing number of studies, and some emerging patterns, there is still scarce data on the immunological consequences of worms-malaria co-infections and the precise mechanisms at play. Nevertheless, vaccine trials and vaccination campaigns in the tropics should definitely take worms into account [48].

More research is needed to disentangle the underlying immunological mechanisms, the differences between different helminths, and the effect of age on the outcome of co-infections. The present review can cautiously conclude that worms, notably *Ascaris*, are associated with protection from severe complications and that they can, notably hookworm, lead to increased malaria incidence or prevalence. Thus, although it is tempting to rush to the conclusion that de-worming patients would reduce malaria, it still seems wise to be attentive to the potential scenario of an increase of severe malaria. Monitoring the incidence of malaria and severe malaria before and after vertical de-worming campaigns seems a minimum test to make sure there is a better understanding of what is going on at a population level, and that, for individual patients, it is best to follow the medical precept *primum non nocere *- first do no harm.

## Competing interests

The authors declare that they have no competing interests.

## Supplementary Material

Additional file 1 **Table S1: Studies on the interactions between worms and malaria in Humans **[49]. Click here for file

Additional file 2 **Table S2. Animal models of coinfection between worms and malaria **[50-72]. Click here for file
