# Supplementary material for: Interactions between worms and malaria: Good worms or bad worms?
Source: Malar J. 2011 Sep 12;10:259. doi: 10.1186/1475-2875-10-259 (PMC3192711; doi:10.1186/1475-2875-10-259)
Supplement: Additional file 1 — Table S1: Studies on the interactions between worms and malaria in Humans [49]. [file 1475-2875-10-259-S1.DOC]

**Additional file 1. Studies on the interactions between worms and malaria in Humans.**

| **Study site date** | **Age group** | **Design &**  **Sample size** | **Worm species** | **Malaria** | **Remarks** |
| --- | --- | --- | --- | --- | --- |
| **Comoro Islands [1]**  **1977** | Children 0-14 | Cross sectional & ecological  869 | *Ascaris lumbricoides* | Decreased prevalence/incidence | It is not clear if prevalence or incidence is considered  The comparison is not with absence of *Ascaris* but between high burden and low burden |
| **Comoro Islands[2]**  **1978** | Children 2-14 | Randomized controlled trial  122 | *Ascaris lumbricoides* | Increased ‘incidence’ between 6-14 days after piperazine treatment | Given the short interval, malaria was probably present but asymptomatic before piperazine |
| **Thailand**  **[3]**  **2000** | Adults | Case control  537 | *Ascaris lumbricoides* | Protection from cerebral malaria  Renal failure  Pulmonary edema | Dose dependent protection  Protection increases with the number of GI species involved |
| **Thailand[28]**  **2001** | Adults | Case control  179 | pooled | Protection from renal failure  Protection from jaundice | Linear trend between egg count and odds of renal failure  Fewer mature schizonts in GI nematode-infected patients |
| **Thailand**  **[33]**  **2001** | Adults | Cross sectional  200 | hookworm | Decreased admission temperature |  |
| **Thailand [49]**  **2001** | Adults | Cross sectional  291 | Pooled (excluding hookworm) | Increased anemia |  |
| **Thailand**  **[36]**  **2001** | Adults | Cross sectional  928 | *Ascaris lumbricoides* | More mixed Pf-PV infections |  |
| **Thailand**  **[22]**  **2002** | Adults | Cohort  731 | Pooled | Increased incidence | Incidence tends (P=0.07) to increase with the number of worm species  Mostly hookworm (57%) significant linear trend between incidence and hookworm egg burden |
| **Thailand [27]**  **2002** | Adults | Case control  384 | pooled | Protection from cerebral malaria | *Ascaris* only individual species significantly associated with protection (AOR=0.15)  Controlling for body mass index |
| **Thailand**  **[34]**  **2002** | Adults | Cross sectional  307 | pooled | Increased gametocyte carriage | Association is confounded by lower hemoglobin counts  Linear trend between egg count and odds of gametocyte carriage |
| **Senegal**  **[23]**  **2003** | Children 1-14 | Cohort  80 | pooled | Increased incidence |  |
| **Thailand**  **[35]**  **2003** | Adults | Cross sectional  248 | *Trichuris trichiura* | Increased multiplicity of infection |  |
| **Senegal**  **[16]**  **2004** | Children | Case control  128 | *Ascaris lumbricoides* | Increased severe malaria | Case definition includes vomiting (exposure can cause vomiting=>bias)  Case classification not performed by physician (39% of the severe malaria diagnoses in fact not malaria)  Controls do not have malaria |
| **Senegal**  **[30]**  **2004** | Children | Cohort  512 | *Schistosoma mansoni* | Increased *falciparum* malaria incidence | No linear trend between egg burden and malaria but heavy worm burdens had highest malaria incidence |
| **Senegal**  **[32]**  **2004** | Children 7-15& adults>30 | Cross sectional  79 children + 49 adults | *Schistosoma haematobium* | No difference in parasitaemia | NB. Patients with clinical mild or severe malaria excluded |
| **Thailand**  **[37]**  **2005** | Adults | Cross sectional  119 | *Ascaris lumbricoides* | Negative correlation between proportion of fertilized *Ascaris* eggs and admission temperature in *vivax* malaria |  |
| **Uganda**  **[4]**  **2005** | Children+Adults | Cross sectional  856 | Pooled  +individually | No association |  |
| **Senegal**  **[26]**  **2005** | Children | Longitudinal  523 | *Schistosoma haematobium*  *GI nematodes* (pooled) | Decreased parasitaemia | Association with decreased parasitaemia observed in low egg burdens  Non significant trend for negative association  For GI nematodes & malaria no association with parasite densities. Pooled but mainly *Ascaris.* |
| **Mali**  **[31]**  **2005** | Children (4-14) | Cohort  676 | *Schistosoma haematobium* | Decreased incidence of *clinical* malaria | IL-6 and IL-10 levels  blunted by *S. haematobium*  [50] |
| **Madagascar**  **[6] 2006** | Children | Randomized controlled trial  350 | *Ascaris* | Increased *falciparum* parasitaemia after levamisole treatment of *Ascaris* in children> 5 years | No apparent effect before 5 years of age |
| **Madagascar**  **[7]2007** | Children | Randomized controlled trial  212 | *Ascaris* | Increased *falciparum* parasitaemia after levamisole treatment of *Ascaris* in children> 5 years | No apparent effect before 5 years of age |
| **Kenya [14]**  **2008** | Children | Cohort  387 | Pooled | No increased incidence |  |
| **Uganda**  **[19]**  **2008** | Pregnant women | Cross sectional  2507 | Hookworm | Increased malaria prevalence | *Mansonella* *perstans* associated with hookworm and malaria |
| **Zimbabwe**  **[20]**  **2008** | Children | Cross sectional  1303 | Hookworm | Increased *falciparum* malaria prevalence | *S. mansoni* also associated with increased *falciparum* malaria prevalence |
| **Kenya**  **[8]**  **2009** | Pregnant women | Cross sectional  390 | *Ascaris lumbricoides* | Lower malaria prevalence | Gravida 2 & 3 |
| **Ethiopia**  **[9]**  **2009** | Children & adults | Cross sectional  458 | Hookworm  *Ascaris lumbricoides*  Pooled | Intensity of hookworm infection correlates with malaria parasitaemia  Lower malaria parasitaemia in *Ascaris* heavy infections  Less severe malaria in helminth- infected persons |  |
| **Zanzibar**  **[25]**  **2009** | Children  6-23 months | Cross sectional+Case control  2322 + 690 | Pooled and individual nematodes | Less malaria in nematode-infected children | Nematode-infected children had higher hemoglobin concentration and mid-upper arm circumference than children without nematodes |
| **Ghana**  **[12]**  **2010** | Pregnant women | Cross sectional  746 | Pooled & individual nematodes | Increased malaria prevalence in hookworm-infected women & *Ascaris*-infected women |  |
| **Senegal**  **[43]**  **2010** | Children  1-14 | Cohort  203 | pooled | Increased malaria incidence | Mostly hookworm (43%)  Then *Ascaris* (10 %) |
| **Brazil**  **[10]**  **2010** | Children 5-14 | Cohort  216 | *Ascaris lumbricoides*  *Trichuris trichiura*  hookworm | Lower drop in haematocrit during *vivax* malaria in patients with *Ascaris*, *trichuris*, or hookworm |  |
| **Thailand**  **[11]**  **2010** | Pregnant women | Cross sectional  829 | hookworm  *Ascaris lumbricoides* | Increased malaria (*vivax* & *falciparum*) in hookworm-infected women  Decreased malaria (*vivax* & *falciparum*) in *Ascaris*-infected women |  |
| **Nigeria**  **[24]**  **2010** | Children  12-59 months | Randomized control trial  320 | All worms | Decreased malaria prevalence on 4-monthly screenings in patients receiving albendazole  Non significant trend to have higher parasitaemia in the placebo group  Non significant increase in haemoglobin concentration in children receiving albendazole | Authors conclude that parasite clearance and immunity may be delayed. *Ascaris* singled out but other helminths also treated by albendazole.  Increased incidence and role of hemoglobin concentration not discussed. |
| **Gabon**  **[13]**  **2010** | Pregnant women | Longitudinal survey  388 | All worms | *Ascaris* associated with increased malaria incidence | Not clear if *Ascaris*-infected women were treated before malaria |
| **Global**  **2010** | Country prevalences for different geohelminths  Malaria incidence data for each country | Classification analysis regression trees/ / Ecological data from 108 countries | *Ascaris lumbricoides*  hookworm | *Ascaris* negatively associated with malaria incidence (10 fold reduction)  Hookworm associated with increased malaria | Ecological data regression analysis trees.  Hookworm effect not observed in multivariable analysis |
| **Uganda**  **[21]**  **2011** | Children & adults | Cross sectional | hookworm | Positive association between Plasmodium and hookworm among preschool-aged children and adults, but not school -aged children.  Spatial and household clustering of coinfections. | Link between malaria and other gastrointestinal nematodes not reported in the study.  Study controlled for socioeconomic and microgeographic factors. |
