# Supplementary material for: Interactions between worms and malaria: Good worms or bad worms?
Source: Malar J. 2011 Sep 12;10:259. doi: 10.1186/1475-2875-10-259 (PMC3192711; doi:10.1186/1475-2875-10-259)
Supplement: Additional file 2 — Table S2. Animal models of coinfection between worms and malaria [50-72]. [file 1475-2875-10-259-S2.DOC]

Table 2. Animal models of coinfection between worms and malaria.

| **Gastrointestinal nematode** | **Plasmodium** | **Outcome (ref)** |
| --- | --- | --- |
| *Heligmosomoides polygyrus* | *Plasmodium yoelii* | Aggravation [50] |
| *Heligmosomoides polygyrus* | *Plasmodium chabaudi* AS | Protection [51] |
| *Heligmosomoides polygyrus* | *Plasmodium berghei*  ANKA | No difference[52] |
| *Heligmosomoides polygyrus* | *Plasmodium berghei*  ANKA | No difference [53] |
| *Heligmosomoides polygyrus* | *Plasmodium chabaudi* AS | Aggravation [54] |
| *Heligmosomoides polygyrus* | *Plasmodium yoelii* | Aggravation [55] |
| *Heligmosomoides polygyrus* | *Plasmodium yoelii* irradiated sporozoites | Suppresses malaria vaccine efficacy[56] |
| *Heligmosomoides polygyrus* | *Plasmodium chabaudi* AS vaccine | Suppresses malaria vaccine efficacy[57] |
| *Heligmosomoides polygyrus* | *Plasmodium chabaudi* AS | Aggravation [58] |
| *Heligmosomoides polygyrus* | *Plasmodium yoeilii* | Reduced vaccine efficacy[56] |
| *Trichinella spiralis* | *Plasmodium berghei* | Protection [59] |
| *Trichinella spiralis* | *Plasmodium berghei* | Protection [60] |
| *Strongyloides ratti* | *Plasmodium berghei* | Protection [60] |
| *Nippostrongylus brasiliensis* | *Plasmodium berghei* | Protection[61] |
| *Schistosoma mansoni* | *Plasmodium yoeilii* | Protection [62] |
| *Schistosoma mansoni* | *Plasmodium yoeilii*  *Plasmodium chabaudi*  *Plasmodium berghei* | No difference [63]  Protection [63] |
| *Schistosoma mansoni* | *Plasmodium yoeilii*  *Plasmodium berghei* | No difference [46] |
| *Schistosoma mansoni* | *Plasmodium berghei* vaccine | Reduced vaccine efficacy  [64] |
| *Schistosoma mansoni* | *Plasmodium berghei* | Aggravation [65] |
| *Schistosoma mansoni* | *Plasmodium chabaudi* | Protection [66] |
| *Echinostoma caproni* | *Plasmodium yoeilii* | Enhanced transmission [67] |
| *Echinostoma caproni* | *Plasmodium yoeilii* | Aggravation [68] |
| *Brugia pahangi* | *Plasmodium berghei* | Protection [69] |
| *Litomosoides sigmodontis* | *Plasmodium berghei* | Protection [70] |
| *Litomosoides sigmodontis* | *Plasmodium berghei* | Protection [71] |
| *Litomosoides sigmodontis* | *Plasmodium chabaudi* | Aggravation [72] |
